# A Framework for Maintenance and Scaling of an Evidence-based Guideline Program

**DOI:** 10.1097/pq9.0000000000000153

**Published:** 2019-03-08

**Authors:** Annie Seneski, Anne M. Stack

**Affiliations:** From the Division of Emergency Medicine, Boston Children’s Hospital, Boston, Mass.

## Abstract

**Introduction:**

Use of Evidence-based Guidelines (EBGs) has been shown to improve and standardize care. After implementation and maturation of a guideline program, next steps include incorporating new evidence, sustaining adherence, minimizing measurement burden and fostering scaling of the program. We propose a framework for maintenance and dissemination of an EBG program.

**Methods:**

Using a program of 28 EBGs developed for use in a pediatric emergency department (ED) in 2010, we developed: a framework for iterative review and revision, a strategy to measure ongoing use in practice and an approach for minimizing repeated measurement sufficient to evaluate outcomes. Also, we created a process to spread the EBG program to the hospital's Department of Pediatrics.

**Results:**

The framework for maintenance and spread of a program of EBGs resulted in an annual review of individual guidelines with 14 revisions warranted by new evidence, some leading to decreased medication utilization and hospitalization rates. We demonstrated adherence to key quality measures, and decreased the number of measures from 89 to 43, retiring 46 measures with stable peformance. We spread the process for program development to the hospital pediatric department resulting in 36 new EBGs.

**Conclusions:**

We developed a framework for maintenance and scale of a program of EBGs. Our key learning points were that regular incorporation of new evidence, assessment and feedback on performance and leadership with administrative support are necessary to maintain improvement. This framework may assure sustainability and inform other guideline programs. We offer processes to promote guideline dissemination within an academic hospital.

## INTRODUCTION

Evidence-based guidelines (EBGs), systematically developed statements to assist practitioner and patient decisions about appropriate healthcare for specific clinical circumstances,^1^ have emerged as a tool to improve and standardize clinical care. EBGs are useful in emergency medicine given the breadth of presenting conditions, stressful environment and need for timely care.^2^ We recently reported on the implementation of an EBG program in a single institution.^3^ Other hospitals have instituted similar programs.^4–8^ Once established and embedded into care, the next challenge for an effective EBG program is the ongoing revision of the EBGs, the sustained use in practice, and development of effective and efficient strategies to minimize unnecessary measurement. There are well-documented strategies to sustain improvement of quality improvement (QI) initiatives, including eliminating causes of noncompliance,^9^ ongoing observations of desired behavior with immediate feedback,^10^ highly reliable interventions, and leadership support.^11,12^ Much of the literature relates to sustaining specific guidelines.^13–15^ We know less about how to sustain a program with many guidelines, including how to determine if the recommendations are embedded in practice and how to reduce the resources needed for improvement and measurement. Another consideration is the spread of guidelines. QI techniques have been used to increase the number of hospitals creating and adhering to guidelines.^16,17^ It has been difficult to ensure that hospitals implement reliable interventions to aid in sustaining the initiatives.^17^

We introduced an EBG program into our hospital’s emergency department (ED) in 2010, and it has grown to include 28 individual EBGs (Table 1). This program has successfully standardized care,^18,19^ decreased resource utilization,^14,20^ and decreased the cost.^14,18,21^ It has been well accepted by clinicians.^3^ The evolution of the program has led to the development of processes for incorporating new evidence and sustaining guideline adherence while minimizing administrative burden. We report on our approach to overcome the challenges of maintaining, growing, and spreading a successful EBG program within the hospital.

## METHODS

**Fig. 1.**
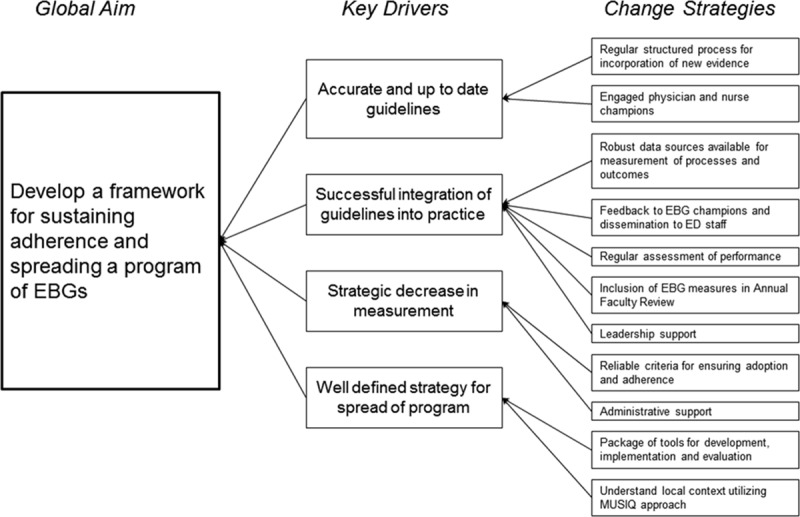
Driver diagram for maintaining and scaling of an EBG program. MUSIQ, Model for Understanding Success in Quality.

To anchor our work, we created a driver diagram (Fig. 1) for maintaining and scaling an EBG program. Provision of a global aim, key drivers, and change strategies helped maintain focus and support the program as a whole.

### Incorporation of New Evidence

**Fig. 2.**
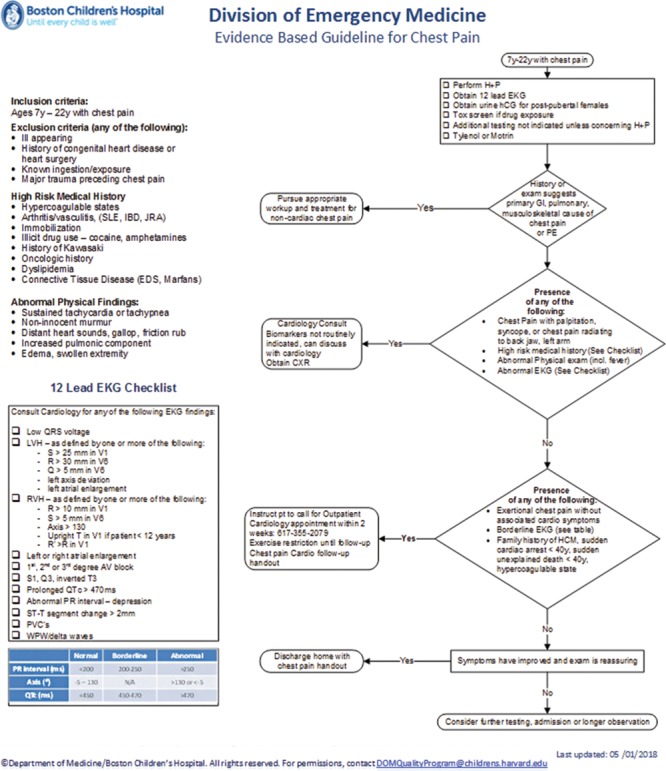
Example of EBG. This guideline was developed for educational purposes only and for use in the Division of Emergency Medicine program at Boston children’s Hospital. Decisions about evaluation and treatment are the responsibility of the treating clinician and should always be tailored to individual clinical circumstances. This image © Department of Medicine/Boston Children’s Hospital. This image was republished with permission of the Department of Medicine/Boston Children’s Hospital. Permission from the copyright holder is required for reuse. EKG, electrocardiogram; hCG, human chorionic gonadotropin; SLE, systemic lupus erythematosus;IBD- inflammatory bowel disease; JRA- juvenile reactive arthritis;EDS- Ehlers-Danlos syndrome;CXR, chest x-ray; GI, gastrointestinal; PE, pulmonary embloism; AV, atrioventricular; PVC, Premature ventricular contraction.

The evidence must be current and accurate to achieve the EBG program’s primary purpose of providing optimal care. In the beginning, we created a process whereby a dedicated physician and nurse champion developed each EBG (see Fig. 2, eg, guideline). To ensure that the guidelines remained current, we developed 2 strategies. First, we select a peer faculty member to perform journal review monthly. If new evidence emerges relevant to an EBG, champions are contacted for further review and revision if warranted. Second, the EBG champions perform a complete literature review annually, and if necessary, propose new evidence for incorporation into the EBG. With regular review of the evidence, we considered the original EBG research framing questions (PICO^22^) and modified as indicated. If new evidence led to substantial changes in the guideline, we consider and track new PICO questions closely. Using this process, we can sustain regular review and ensure modifications are incorporated as needed. New evidence is evaluated using Grading of Recommendations, Assessment, Development, and Evaluations criteria (gradeworkinggroup.org).^23^ Subsequently, the evidence is vetted with the ED physicians at faculty conference to gain awareness and consensus, and then revisions are incorporated into the guideline. We disseminate the revised EBG to clinicians through presentations at meetings, individual education, e-mails, posters, and bulletin boards. We maintain an intranet webpage for easy access and a direct link to condition-specific order sets in the electronic medical record (EMR). Our prior work presented data on successful uptake strategies, and note that paper copies of EBG were more useful for nurses and electronic resources more useful for physicians.^3^

New candidate measures reflecting new recommendations may be proposed by the EBG champions and program leaders. These measures are vetted for feasibility, usability, and importance by the ED EBG leadership team and champions depending on data capabilities and clinical relevance, and then monitored monthly to ensure changes have become embedded in practice.

### Sustaining Adherence

Even for EBGs without substantive content changes, sustained adherence to the guideline is necessary for the success of the program. During the development of a new EBG, we choose 3–5 key measures for tracking. Ideally, these are outcome measures that indicate if the guideline has improved care. Often in an emergency setting, due to the brevity of patient interaction, we use key process measures to track adherence. Some institutions report tracking order set use to measure guideline access. We felt it was more relevant to directly measure adherence to process measures and outcomes, such as computerized tomography (CT) use for patients with suspected appendicitis. After approximately 25 periods of baseline data are gathered, these measures for implemented EBGs are tracked frequently. The nature of the condition and volume determines frequency (eg, monthly or weekly). Data are generated electronically from the data warehouse (Microstrategy, Tysons Corner, Va.) fed by the EMR (Cerner Corporation, Kansas City, Kans.). A local ED EBG leadership team comprised 2 data analysts with 3–5 years of healthcare experience, a QI consultant, and physician quality leader meet weekly to review data and statistical process control charts for a group of guidelines on a rotating basis. Trends and shifts^24^ in the data are discussed, and measures that need refinement or have been stable for an extended period. We then relay this information to the physician champion for review and recommendations. These regular meetings support early detection of lapses in performance and allow the champions to take steps to improve performance. Interventions to boost compliance range from e-mail reminders and educational campaigns to reintroducing active QI methodology depending on the degree of the slide in performance. If new measure targets are not met within a few weeks or months, depending on the visit frequency of patients with the presenting condition, the EBG champions, with guidance from the EBG leadership team at the divisional or departmental level, provide booster doses of awareness via group data sharing, emails, individual feedback, and posters until we establish adoption.

One such method to embed EBG adherence is the inclusion of physician performance in annual faculty reviews.^25^ Each year we incorporate selected EBGs into the review process. These are chosen based on the strength and importance of the guideline recommendation, such as antibiotic stewardship, resource utilization, and patient centeredness. Although not directly linked to monetary risk, individual physician performance is reviewed with the physician and compared with the performance of peers.^25–27^

### Rightsizing Measurement

**Fig. 3.**
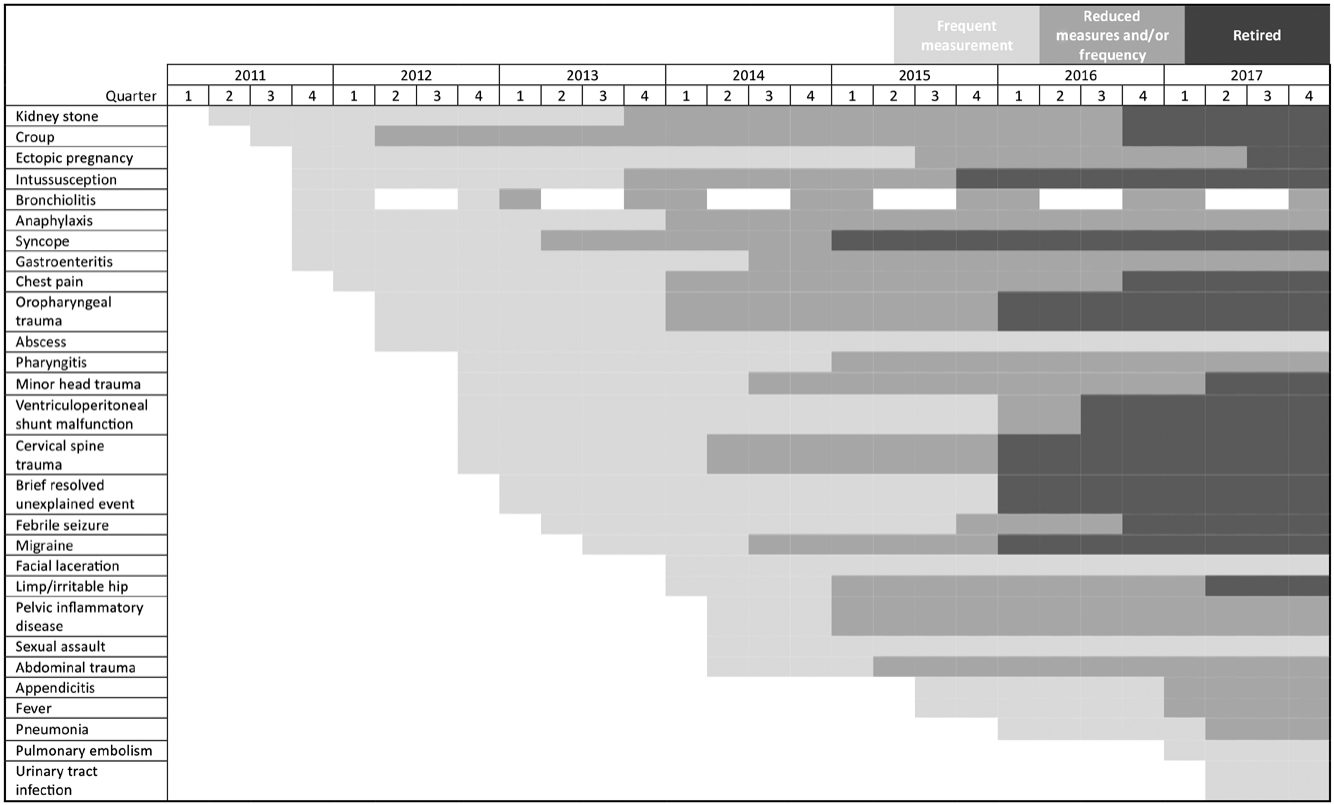
Timeline of reduction in EBG measurement frequency

EBG measures are defined using a standard measurement plan that is completed jointly by the ED EBG leadership team and ED EBG champions.^3^ These plans outline inclusion and exclusion criteria, data source, diagnostic codes, and detail on numerator and denominator, risk stratification as needed, display format, and frequency of review. Once performance stabilizes at a target level for a sustained period (eg, 6 months), we reduce the frequency of measurement (Fig. 3). Achieving a preset target consistently, or steadily shifting mean performance toward optimal, triggers to reduce monitoring frequency. Stability requires achievement of minimal data variation, which is strongly influenced by the number of cases; therefore, less frequent diagnoses may mandate longer surveillance periods.

The timeline for reducing measurement also varies based on the criticality. Rarer and/or critical guideline conditions, such as pediatric pulmonary embolism, are measured for a longer duration to ensure incorporation into practice. Likewise, guidelines for more routine and/or less acute conditions, such as acute gastroenteritis, can be moved to less frequent measurement cycles more rapidly. This modification allows the team to put focus on areas that need improvement or are most important for successful outcomes. After a period of consistent performance (generally 6–12 months), spot checks are performed every 6–12 months. If periodic audits reveal a decline in performance, measures return to a regular cycle of review to boost adherence. When performance is consistently stable at target, the measurement is retired. At this point, we are confident that the guideline is embedded into routine practice and considered standard care.

### The Spread of a Program of Evidence-based Guidelines

**Fig. 4.**
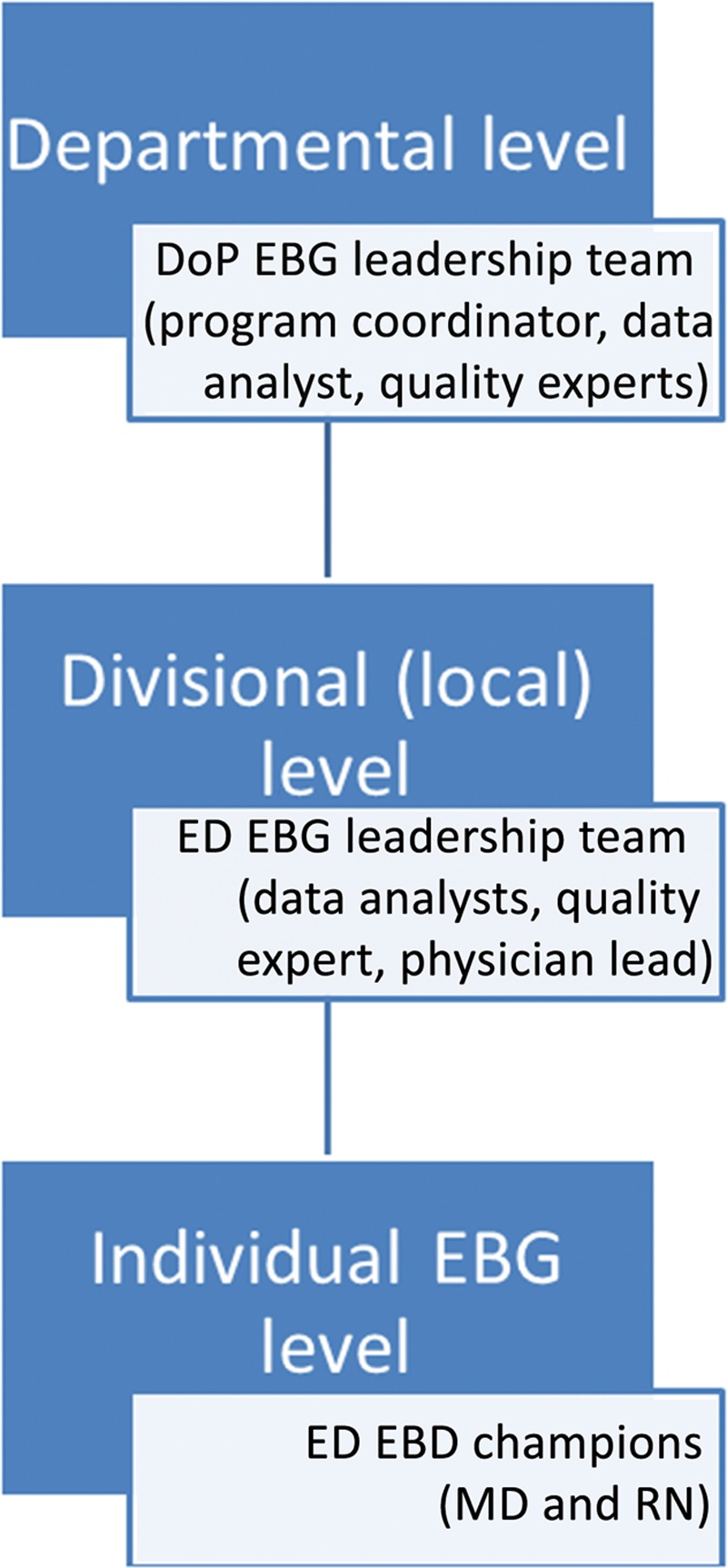
EBG governance structure. DoP, Department of Pediatrics; MD, doctor of medicine; RN, registered nurse.

Spreading a successful EBG program is important for influencing the best practice. We collaborated with departmental quality leaders to share the strategy and tools for development and implementation of the EBG program.^3^ The ED EBG leadership team was available as a resource and continues to consult as needed. The departmental EBG team, similar in make-up to the ED team, included a data analyst, QI consultants, pharmacist, and physician leader. They met regularly with subject matter experts in the department who served as local champions (see Fig. 4 for governance structure). Modifications at the department level included requirement for a detailed list of the relevant literature, changes in the measurement plan templates, and modifications in the implementation process with adaptations to the local context (**see example**, **Supplemental Digital Content 1**, which displays EBG buy-in worksheet, http://links.lww.com/PQ9/A75). Individual physician performance was measured either by chart review or database extraction. The departmental quality program supported this collaboration with a small amount of funding ($5,000 per guideline). We also developed a mobile app to facilitate access to guidelines.

According to the policy at Boston Children’s Hospital, this work met criteria for operational improvement activities and not human subject research. Therefore, review and approval by the institutional review board were not required.

## RESULTS

### Incorporation of New Evidence

Through active participation by guideline champions, supported by the quality administrative team, all 28 guidelines have been reviewed and/or revised at least annually, and 14 have undergone major revisions. Successful incorporation of new evidence has led to decreased resource utilization. One such example is reduced hospitalization rates for patients with idiopathic intussusception from 92.3% in 2010 to 32.4% in 2016 after new data on the safety of ED discharge after air contrast enema reduction was published.^26^ Albuterol administration for infants with bronchiolitis decreased after new American Academy of Pediatrics guidelines recommending against its routine use were released in November 2014. The initial rate of albuterol use was 53%, which dropped to 43% within a month after new guideline implementation, but it was not until we formally revised the local EBG in November 2015 with new recommendations that the rate dropped to 18%. The anaphylaxis EBG was introduced in November of 2011 and led to initial avoidance of 140 hospital admissions by December 2014.^19^ After we incorporated new evidence^27^ in June of 2015, the hospitalization rate fell even further, avoiding an additional 32 hospital admissions by November 2017.

### Sustaining Adherence

**Fig. 5.**
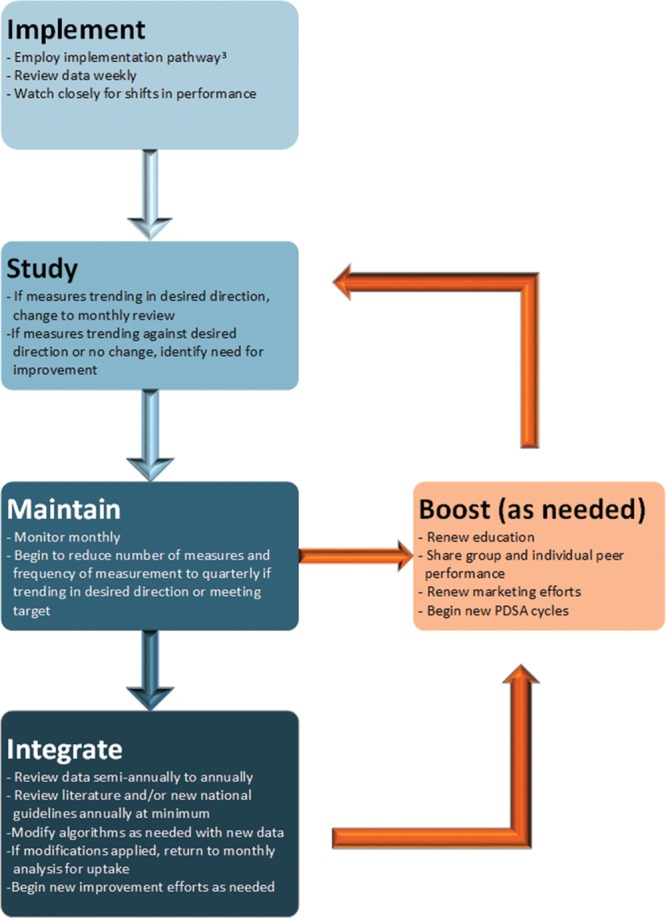
Framework for measurement strategy for a program of EBGs. PDSA.

We developed a new framework for tailoring measurement and ensuring guideline establishment into practice (Fig. 5). Through regular examination of performance on key quality measures, formal annual individual faculty review with ED leadership and robust attention to keeping the guidelines current by EBG champions, suboptimal adoption has been improved and performance at target sustained. For example, our facial laceration EBG recommended the first dose of antibiotics for dog bites should be administered before discharge from the ED. After initial introduction, the guideline raised performance aided by inclusion in annual faculty reviews and sharing of peer performance, but it required 2 years of individual provider feedback to attain the target level of performance. Using this process, we detected a decline in performance for patients with pelvic inflammatory disease receiving recommended antibiotics. Because the condition was infrequent, we added individual provider feedback on “missed” cases for 3 years after initial implementation and have now demonstrated 100% compliance over the past several months. Currently, we measure 6 EBGs monthly, 7 quarterly, and 15 have been retired or have annual or biannual spot checks.

### Rightsizing Measurement

Using our framework, we have decreased regularly monitored EBG performance measures from 89 to 43 while maintaining high performance and incorporating new guidelines and associated measures. Seventy-eight percent of EBGs have met goal performance. We have removed these EBGs from active monitoring. For example, the introduction of the minor head trauma EBG significantly decreased the hospitalization rates for patients with isolated nondepressed linear skull fracture.^14^ From its implementation in October 2012 through September 2017, we have safely avoided over 90 hospital admissions. Although we communicate success in program overviews, measurement occurs biannually, and there is no ongoing active improvement supporting the guideline as we consider it embedded into standard practice.

Many other successes have led to reduced measurement. The change to use magnetic resonance imaging (MRI) as the imaging modality for patients evaluated for ventricular shunt malfunction is another example. The percent compliance with the imaging recommendation increased from 44% to over 80% with the introduction of the EBG. After 17 months of high performance, we retired the measure. Subsequent annual spot checks ensured we still followed the recommendation.

We also identified cases where performance declined after less frequent measurement (eg, completion of scores for patients with gastroenteritis). After a 6-month spot check identified the performance degradation, we reinitiated active monitoring.

### The Spread of a Program of Evidence-based Guidelines

We spread the process to non-ED settings in the Department of Pediatrics including ambulatory and inpatient units. Over 4–5 months, we shared our expertise and the tools for development and implementation of the EBG program with the departmental EBG leadership team. We supported the development of 36 new EBGs (**Supplemental Digital** **Content 2**, which provides list of departmental EBGs, http://links.lww.com/PQ9/A76). They range from straightforward and common conditions, such as management of acute otitis media, to more rare conditions, such as evaluation and treatment of Ehlers–Danlos Syndrome. All departmental EBGs are currently undergoing measurement and analysis in a process similar to the ED to determine their impact on care. Also, we have shared EBGs with all hospital providers through links to an online intranet site. This portal allows for easy access to all guidelines across the enterprise.

A novel approach to foster ease of access and spread of guidelines has been the development of an EBG mobile app (EBGs, Boston Children’s Hospital). The app is downloadable onto both iOS and Android mobile devices. It has been downloaded 281 times, with 101 monthly users. On average, the app has been used 9 times a day since go-live in November 2017. It includes step-by-step decision support mirroring the ED EBG algorithms and is updated with all EBG revisions.

## DISCUSSION

We present a practical process for sustaining and spreading a program of EBGs. The major lessons learned were as follows:

1. A regular, structured process for incorporation of new evidence is critical to maintain the guidelines as “living documents” and thus the trust of those who use them.
2. We achieved sustainment of EBGs in practice through regular assessment of performance, dedicated feedback to stakeholders, and strong leadership support.
3. Consistent leadership and administrative support are needed to maintain a successful EBG program and to assess uptake for measurement reduction.
4. The internal spread of the EBG program is achievable through sharing a package of tools for development, implementation and evaluation, practical support, and acknowledgement of challenges presented by a new context.

For an EBG program to flourish, it is important that physicians trust the process and believe in its integrity.^28^ Through annual, at a minimum, literature reviews by EBG champions and thoughtful vetting of new evidence with colleagues, we ensure that the documents remain fresh and reliable.

As we learned in the EBG program development stage, strong leadership support is essential for continued success. Leadership-led annual faculty reviews of individual physicians emphasize the program’s value, especially when we provide peer comparison. Also, resources for data teams, QI experts, and dedicated QI physician time are critical to success. Without this, there would be limitations to the regular cycle of performance and feedback which we found fundamental to the sustainment of the program. We needed administrative support for data extraction, project management, QI, and regular review of performance. These resources can also support the development and maintenance of physician performance portfolios which we have noted to be an important component of faculty development.^25^

Periodic assessment of performance is fundamental to QI. Our framework for decreasing the frequency of assessments and ultimate retirement of measurement has led to efficiencies in administrative and clinician resources. Strategies for measuring retirement include achievement of a ceiling effect or sustained performance at or above target.^29^ In our program, we sought sustained performance at the target to judge retirement eligibility.

The spread of the program to other areas of the hospital was relatively seamless as shown by the development of a large number of regularly accessed guidelines.^30^ There are several factors to consider when spreading to a new context, including those outlined in the Model for Understanding Success in Quality.^31^ These include the external and organizational environments, senior leadership support, the local QI culture, and the QI team with its skill and motivation. The context at the department level was in many ways similar to the ED concerning the environment and data sources. The QI expert teams were similarly engaged and trained. One substantial difference was that leadership in each division offered varying support and the EBG leadership team at the departmental level was tasked with navigating different quality and subject matter expert “microenvironments.” The leadership team overcame these contextual barriers by soliciting advice from the subject matter experts to determine the need for a top-down versus consensus approach. In this way, the local subject matter experts guided paths to buy-in.

The interactive mobile app allows readily available access and assistance in spreading guideline adoption. Rotating residents report benefitting from the ready availability of standardized care guidelines on their phones and other devices which they also use for patients in other EDs.

There are limitations to consider when developing and sustaining an EBG program. We had strong ED leadership support and institutional resources that may not be generalizable to other settings. We had well-developed technology, including the ability to easily access EMR data and create modifications to specific elements such as order sets.

## CONCLUSIONS

We propose a framework for maintaining a program of EBGs with strategies to reduce measurement. We demonstrate that it is possible to successfully spread an ED EBG program to other areas of a hospital. We believe adoption of this method of sustainment and spread of an EBG program are possible in similar settings.

We acknowledge Taruna Banerjee, MPH, and the DoP Quality team for their assistance with the spread of the EBG program.

## DISCLOSURE

The authors have no financial interest to declare in relation to the content of this article.
